# Clinical, cytogenetic, and molecular findings in a patient with ring chromosome 4: case report and literature review

**DOI:** 10.1186/s12920-019-0614-4

**Published:** 2019-11-21

**Authors:** César Paz-y-Miño, Ana Proaño, Stella D. Verdezoto, Juan Luis García, Jesús María Hernández-Rivas, Paola E. Leone

**Affiliations:** 1 Centro de Investigación Genética y Genómica, Facultad de Ciencias de la Salud Eugenio Espejo, Universidad UTE. Av. Mariscal Sucre y Av. Mariana de Jesús, Sede Occidental, Bloque I, 2 floor, 170129 Quito, Ecuador; 2 Institute of Molecular and Cellular Biology of Cancer (IBMCC), University of Salamanca-SACYL-CSIC, Salamanca, Spain; 3 Molecular Medicine Unit, Department of Medicine, Biomedical Research Institute of Salamanca (IBSAL), Salamanca, Spain; 4 Servicio de Hematología, Hospital Universitario de Salamanca, Universidad de Salamanca, Salamanca, Spain

**Keywords:** 46,XX,r(4)(p16.3q35.2), Ring chromosome 4, Mosaic, inv dup del rearrangement, FISH, Mapping array

## Abstract

**Background:**

Since 1969, 49 cases have been presented on ring chromosome 4. All of these cases have been characterized for the loss of genetic material. The genes located in these chromosomal regions are related to the phenotype.

**Case presentation:**

A 10-year-old Ecuadorian Mestizo girl with ring chromosome 4 was clinically, cytogenetically and molecularly analysed. Clinical examination revealed congenital anomalies, including microcephaly, prominent nose, micrognathia, low set ears, bilateral clinodactyly of the fifth finger, small sacrococcygeal dimple, short stature and mental retardation. Cytogenetic studies showed a mosaic karyotype, mos 46,XX,r(4)(p16.3q35.2)/46,XX, with a ring chromosome 4 from 75 to 79% in three studies conducted over ten years. These results were confirmed by fluorescence in situ hybridization (FISH). Loss of 1.7 Mb and gain of 342 kb in 4p16.3 and loss of 3 Mb in 4q35.2 were identified by high-resolution mapping array.

**Conclusion:**

Most cases with ring chromosome 4 have deletion of genetic material in terminal regions; however, our case has inv dup del rearrangement in the ring chromosome formation. Heterogeneous clinical features in all cases reviewed are related to the amount of genetic material lost or gained. The application of several techniques can increase our knowledge of ring chromosome 4 and its deviations from typical “ring syndrome.”

## Background

Ring chromosomes are rare genetic anomalies in humans that result from the breakage in both ends of a chromosome with the subsequent fusion of the broken ends. This breakage can occur through several cytogenetic mechanisms: breakage in one or both arms of the chromosome with the subsequent fusion of the broken ends or an inversion-duplication-deletion rearrangement (inv dup del) [1–3], leading to loss and gain of genetic material and altered phenotypes. On the other hand, without loss of genetic material, a complete ring chromosome can be formed by fusion of subtelomeric segments or telomere-telomere fusion [4, 5].

Usually, the phenotype of the cases with ring chromosome 4, r(4), presents low birth weight, growth retardation and microcephaly. In addition, there are many features similar to those in Wolf-Hirschhorn Syndrome or similar to cases in which a partial deletion of the short arm of chromosome 4 is observed.

Since 1969, ring chromosome 4 has been described in 49 cases with different clinical findings depending on the amount of genetic material lost in the ring [3, 5–51].

In this report, we present the clinical, cytogenetic and molecular findings of a 10-year-old female patient with a ring chromosome 4 after the third follow-up study.

## Case presentation

The proband is a 10-year-old Ecuadorian girl. According to the ancestry profile of the Ecuadorian population, she is a Mestizo with 72% Native American, 25% European and 3% African ancestry. She is the third child of healthy and non-consanguineous parents. Both parents were 22 years old at the time of her birth. There was an abortion threat at the second month of pregnancy and urinary tract infection during the first trimester of the pregnancy. The patient was born by caesarean section at 40 weeks of gestation with an APGAR of 8–9, weight of 2940 g (below the 10th percentile), length of 47 cm (below the 10th percentile), and head circumference of 32 cm (below the 5th percentile). Upon physical examination, she showed sufficient suckling, good gastric tolerance and loud crying. Additionally, she displayed microcephaly, beaked nose, micrognathia, low-set ears, bilateral clinodactyly of the fifth finger and a 0.5 mm sacrococcygeal dimple. At the age of eight months, magnetic resonance imaging with contrast of her lumbar spine revealed a small fistulous tract that communicated the sacral region with the coccyx; in addition, the presence of lipoma, tethered spinal cord and meningocele was discarded. At the age of two, a computerized axial tomography scan with three-dimensional images of her brain was performed without showing any defects.

Cultures of peripheral blood lymphocytes were taken according to standard techniques. A hundred metaphases were studied in the patient. Patient’s karyotype ten days after birth revealed a mosaicism with ring chromosome 4: mos 46,XX,r(4)(p16q35)[65]/45,XX,-4[5]/46,XX,dic r(4)[5]/46,XX[20]. At the age of five, a second cytogenetic analysis was performed, revealing the following karyotype: mos 46,XX,r(4)(p16q35)[66]/45,XX,-4[6]/46,XX,dic r(4)[5]/46,XX[16].

**Fig. 1:**
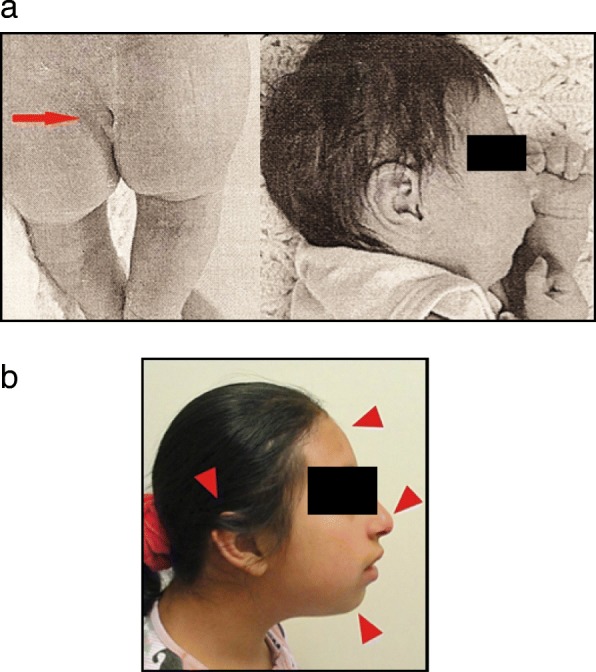
Patient photograph **a**. Patient (ten days after birth). Clinical features, beaked nose, micrognathia, low set ears and a small sacrococcygeal dimple. **b** Patient (ten years old), side view

Patient follow-up included a third clinical and cytogenetic evaluation at the age of 10. Her physical examination showed a height of 125 cm (below the 5th percentile) and a weight of 35 kg (below the 75th percentile). Additionally, she presented mental retardation and a pleasant personality (Fig. 1).

**Fig. 2:**
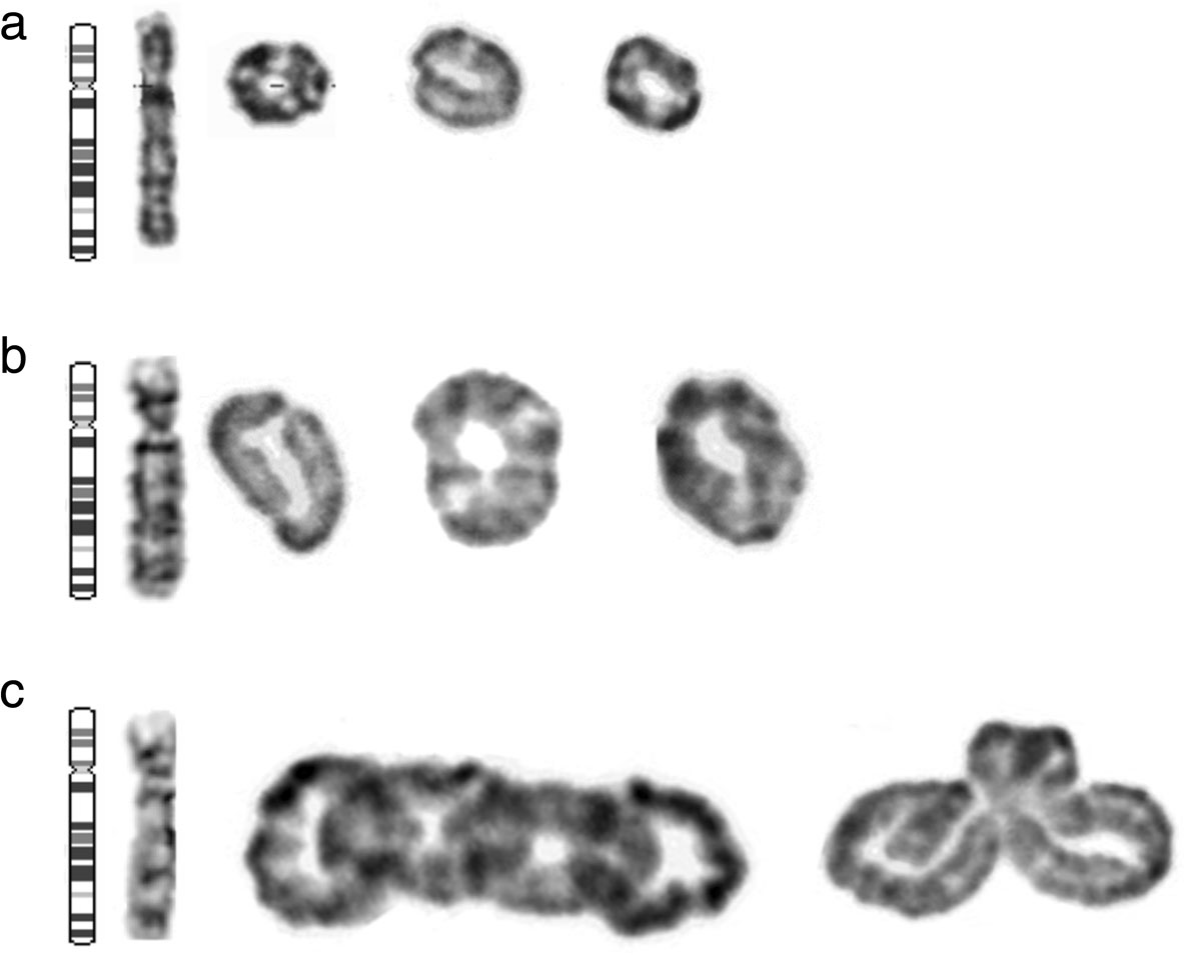
Different types of rings found in the patient. **a**, **b**, **c** Partial G banded karyotypes of peripheral blood lymphocytes showing normal chromosome 4, ring chromosome 4, dicentric ring chromosome 4 and interlocked ring chromosome 4

The karyotype was refined to mos 46,XX,r(4)(p16.3q35.2)[58]/45,XX,-4[7]/46,XX,dic r(4)[7]/46,XX[10] (Fig. 2).

The parents’ karyotypes were normal, 46,XX and 46,XY.

**Fig. 3:**
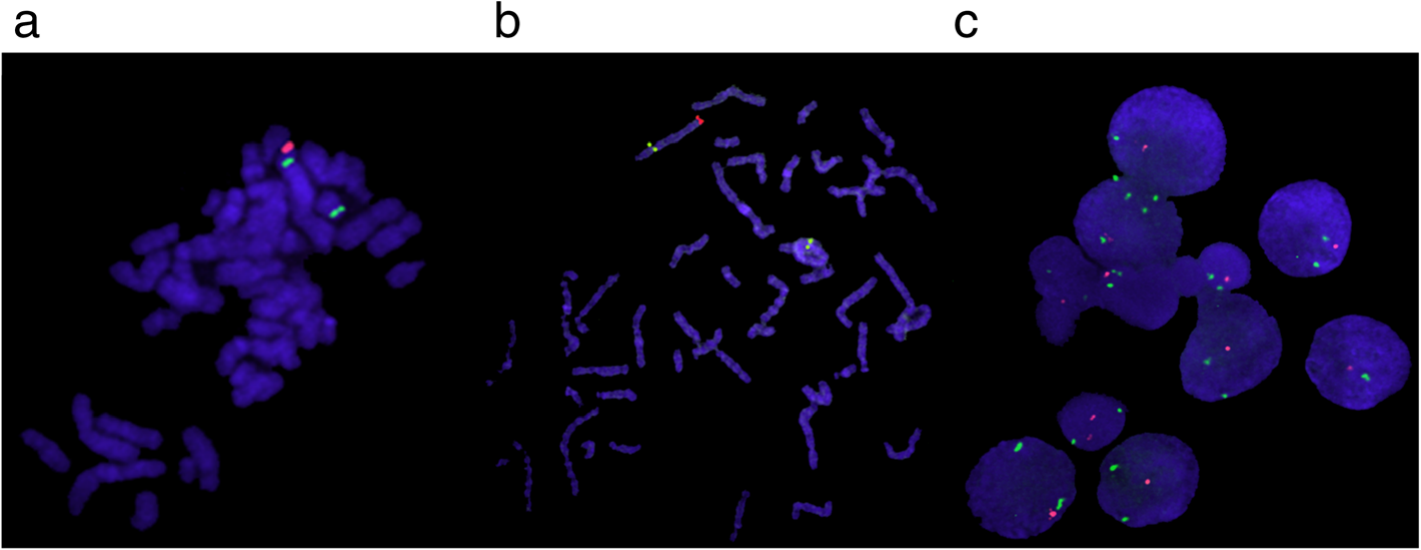
FISH findings in chromosome 4. **a** Patient’s metaphase that shows two green signals corresponding to the centromere region and one red signal corresponding to 4p16.3 region. **b** Patient’s metaphase that shows two green signals corresponding to the centromere region and one red signal corresponding to 4q35.2 region. **c** Green signals are shown corresponding to the centromere region and a red signal corresponding to 4q

Fluorescence in situ hybridization (FISH) analysis was performed on the patient’s culture to confirm the cytogenetic findings. Specific probes were used for the regions 4p16.3 (Chr4: 492,870–793,359), 4q35.2 (Chr4: 190,183,811–190,408,149) and for the human chromosome 4 centromere probe (Chr4: 48,461,959–49,066,696). All probes were purchased from Agilent and used according to the manufacturer’s instructions (Agilent, USA). The patient’s results showed 46,XX,r(4).ish r(4)(p16.3q35.2)(492,870–793,359-,190,183,811–190,408,149-). The chromosome 4 centromere probe showed two hybridization signals. The 4p and the 4q probe showed one signal in each region (Fig. 3).

We decided to determine the status of the chromosomal regions involved in ring formation with single nucleotide polymorphism (SNP) arrays. SNP mapping array was performed using the CytoScan 750 K Cytogenetics Array (Affymetrix, Santa Clara, CA, USA) following the manufacturer’s instructions (Affymetrix, Santa Clara, CA, USA). This analysis showed terminal deletions in both arms of chromosome 4. In 4p16.3, the array showed a 1,710,458 bp deletion, losing one copy of the 34 genes located in that region (from 68,345 bp to 1,778,803 bp). The analysis also exhibited an additional segment of 342,143 bp (from 1,784,441 bp to 2,126,584 bp). Thus, gaining three copies of the nine genes. In 4q35.2, the array showed a 3,056,579 bp deletion (from 187,900,881 to 190,957,460 bp), losing one copy of the seven genes (Table 1).

**Table 1:** Panel of ring chromosome 4 affected genes in the present case

Chromosome region	Pair of bases	Genetic alteration	Number of copies	Genes
4p16.3	68,345-1,778,803	Loss	1	*ZNF595, ZNF718, ZNF876P, ZNF732, ZNF141, ABCA11P, ZNF721, PIGG, PDE6B, ATP5I, MYL5, MFSD7, PCGF3, LOC100129917, CPLX1, GAK, TMEM175, DGKQ, SLC26A1, IDUA, FGFRL1, RNF212, TMED11P, SPON2, LOC100130872, CTBP1, C4orf42, MAEA, KIAA1530, CRIPAK, FAM53A, SLBP, TMEM129, TACC3*
4p16.3	1,784,441-2,126,584	Gain	3	*FGFR3, LETM1, WHSC1, SCARNA22, WHSC2, MIR943, C4orf48, NAT8L, POLN*
4q35.2	187,900,881-190,957,460	Loss	1	*ZFP42, TRIML2, TRIML1, LOC401164, FRG1, TUBB4Q, FRG2*

## Discussion and conclusions

Ring chromosome formation has been described by different mechanisms [1–5]. The ring chromosome 4 in our patient presents a terminal deletion with duplication resulting from inv dup del rearrangement [52]. This deletion and duplication is caused by homologous recombination between duplicated segments near the breakpoints, allowing telomere healing and telomere capture with the formation of an intermediate dicentric chromosome [2, 52].

The influence of deletions has been described in cases of ring chromosome 4. Deletions and duplication could influence the physical characteristics in our patient. To increase our understanding of the effect of deletion, we compared the phenotype of the patient with that of each of the 49 cases previously reported: 20 female and 29 male infants, diagnosed between 21 weeks of gestation and 27 years (Additional file 1).

From all reported physical features, we analysed 53 that are present in at least two patients. Three characteristics were identified to be more common in the 49 cases with ring chromosome 4: low birth weight with 78% appearance frequency, growth retardation (94%), and microcephaly (80%). The less frequent appearance traits include malformed ears (43%), mental retardation (41%), micrognathia (37%), clinodactyly (37%), hypospadias (36% of boys), heart defects (35%), retarded bone age (35%), skeletal abnormalities (33%), hypoplasia postaxial and thumbs alterations (31%), cryptorchidism (29%), short stature (27%), hypertelorism (27%), cleft palate (24%), broad nose (22%), transverse palmar crease (22%), changes in skin (22%), down-turned mouth (20%), seizures (20%), low set ears (18%), epicanthal folds (18%), abnormal phalanges (18%), renal malformation (18%), beaked nose (16%), hypotonia (16%), genitalia abnormalities (16%), low ridge count (16%), clubbed feet (16%), short philtrum (14%), high arched palate (14%), toes alterations (14%), and sacral dimple (12%), ptosis palpebrae (12%), cleft lip +/− palate (12%), prominent glabella (10%), brachycephaly (8%), antimongoloid slant of the palpebral fissures (8%), overlapping toes (8%), intestinal malformation (8%), high forehead (6%), exophthalmos (6%), coloboma (6%), preauricular pit (6%), flat nasal bridge (6%), prominent bridge (6%), teeth deficient in enamel (6%), short neck (6%), other visceral alterations (6%), prominent occiput (4%), strabismus (4%) and hyperactivity (4%).

Our case presented only 8 of these 53 features: microcephaly, mental retardation, micrognathia, clinodactyly, short stature, low set ears, beaked nose and sacral dimple (Additional file 1).

**Fig. 4:**
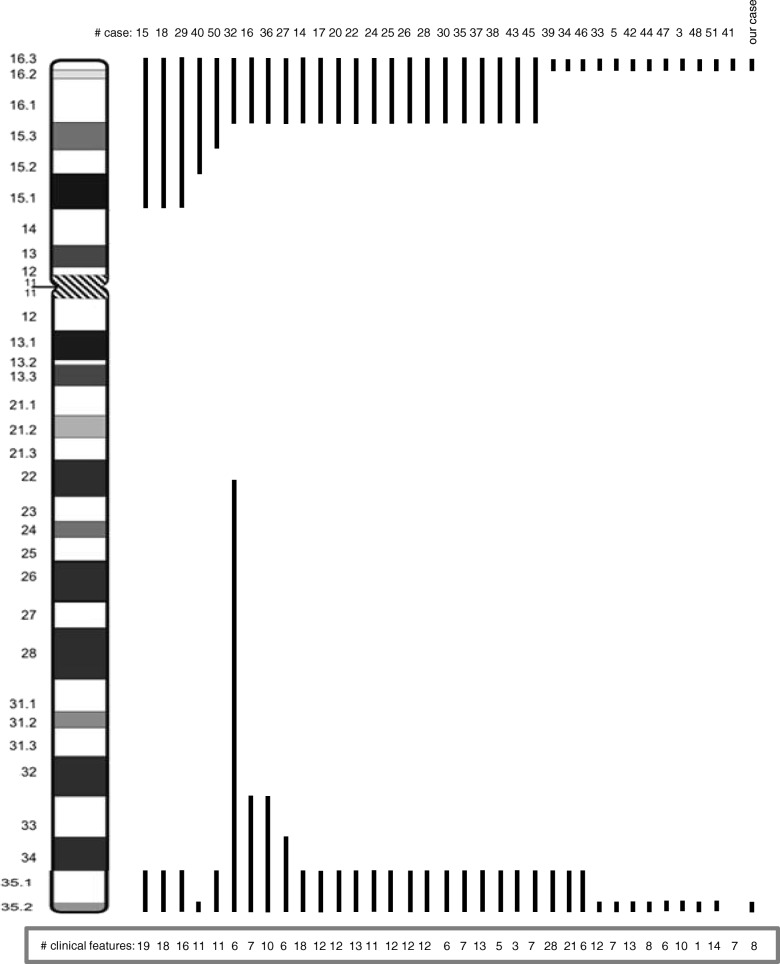
Review of deletions of chromosome 4 by cytogenetic analysis. The position of deletion in each patient is shown as black vertical lines to the right of the ideogram. Each number on the lines corresponds to the bibliographic reference

Analysing closely the reported cases with ring chromosome 4, it is noticeable that increases in the clinical features are present according to the breakpoint. On short arm 4p15, the median number of present traits is 16 (range, 11–19). On short arm 4p16, the median is 11 (range, 3–21), and on 4p16.3, the median is eight characteristics (range, 1–28). On the long arm, the median numbers of characteristics are as follows: on 4q22.3–34, eight (range, 6–10), on 4q35, 12 (range, 3–28), and nine characteristics (range, 1–14) were identified when the breakpoint was on 4q35.2 (Fig. 4).

The association between clinical features and alterations of chromosome 4 was discussed by Parker et al., who described physical findings for the deletion of the short and long arms and ring chromosome 4. Some characteristics were not exhibited in all cases with rings, possibly because they had different breakpoints [13]. In addition, with the exception of 3 characteristics, 18 coincide with Finley’s analysis in relation to deletions of 4p15 and p16 [22], and with the exception of 2 features, 22 coincide with Halal and Vekemans’s clinical and cytogenetic comparison [28].

In our case, duplication of 4p16.3 was found. Several cases have been reported on microduplications with shared clinical features, some of which are associated with microdeletion syndromes [53–56]. Roselló et al. reported a de novo case with a 1.3 Mb deletion and a 1.1 Mb duplication of the terminal 4p region; the patient presented hypertelorism, epicanthal folds, broad nasal bridge, low set ears, and absence of seizures. On the other hand, our case exhibited a 1.7 Mb deletion and a 0.3 Mb duplication, the individual only shows low set ears. Even though our case presented a microdeletion and a microduplication with ring formation which showed less clinical features than the case of Roselló et al. without ring formation, the phenotype may be due to the amount of genetic material altered.

Both cases showed a duplication of *WHSC1*, *WHSC2*, and *LETM1* that can be the cause of clinical features different from Wolf-Hirschhorn Syndrome (WHS) and 4p trisomy syndrome [57]. As remarked by other authors, microdeletions or microduplications on *LETM1* may suggest a cause for the clinical signs of seizures [57]; our case presents microduplications, and seizures remain absent. Microduplication syndrome also describes clinical features, such as psychomotor and language delay, seizures, high forehead with frontal bossing, hypertelorism, prominent glabella, long narrow palpebral fissures, short neck and low set ears [56], where only the latest characteristic is present in our case.

The cytogenetic results of our patient showed 79% of cells with rings in different presentations: cells with 46 chromosomes with monocentric ring, dicentric ring, polyploid cells with monocentric ring, and interlocked ring. The rest of the cells had a normal karyotype, cells with loss of chromosome 4 and cells polyploid with an absent ring. Karyotype comparison with 49 reported cases is presented in Additional file 2, where 37 cases detailed the ring chromosome 4 in mosaic state. Twelve cases described the presence of a ring without any specificity regarding whether it was in a mosaic state.

**Fig. 5:**
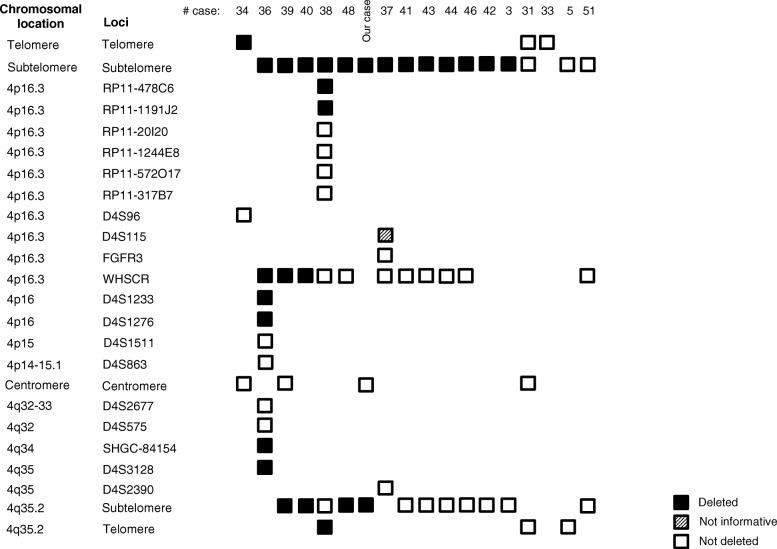
Review of deletions of chromosome 4 by FISH. Each number corresponds to the bibliographic reference

Of the 49 cases reported, only 17 made reference to the use of FISH analysis [3, 5, 31, 33, 34, 36–44, 46, 48, 51]. Although four cases presented normal results, this does not exclude the possibility that the ring chromosomes may have lost material in one or two arms. In the other fourteen cases, including our patient, losses were found in 4p16. Ten cases with terminal deletions (one in telomere and nine in subtelomeres), three cases with deletions at 4p16.3 (one with a breakpoint distal to RP11-20I20 and two with deletion of the Wolf-Hirschhorn Syndrome critical region) and one case with deletion at 4p16 (with a breakpoint distal to D4S1511 at 4p15). Six cases showed losses in 4q, one case in 4q34 (with a breakpoint distal to D4S575) and five cases with terminal deletions (one in a telomere and four, including our patient, in subtelomeres). The results of FISH analysis have allowed us to define or confirm the breakpoints of the ring chromosomes identified by cytogenetic analysis. Cases with ring chromosome 4 show frequent loss of subtelomeric segments (Fig. 5).

To refine the length of the losses of chromosome 4 in our patient, we used a mapping array. A deletion in 4p between 68,345 bp and 1,778,803 bp and a deletion in 4q between 187,900,881 bp and 190,957,460 bp were detected, refining the original information of losses by FISH between 492,870 bp and 793,358 bp, and 190,183,811 bp and 190,408,149 bp, respectively. Additionally, a gain of 342,143 bp of 4p16.3 between 1.78 and 2.12 Mb was found to contain 9 genes. The loss of some of these genes has been associated with WHS. However, as in our case, the gain of these genes as has already been explained can show different clinical features.

**Fig. 6:**
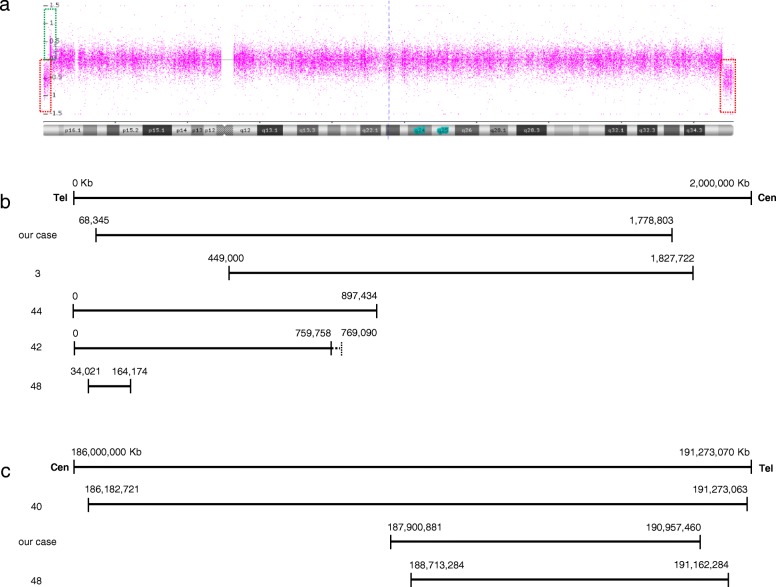
Review of deletions of chromosome 4 by Arrays. **a** The mapping array plot is shown as copy number (Y-axis) versus cytogenetics co-ordinates (X-axis). The deletions were identified (red dashed boxes) in 4p16.3 (1.71 Mb) and 4q35.2 (3.06 Mb) and the gain (green dashed box) in 4p16.3 (342 kb). **b** The first line represents 4p16.3 from 0 to 2,000,000 bp, the following lines show the loss location in our case and in other cases reported in the literature (each number on the left corresponds to the bibliographic reference), the solid line represents the deletion and the dotted line indicates the region that may or may not be deleted. **c** The first line represents 4q35.2 from 186,000,000 to 191,273,070 bp, the following lines show the loss location in our case and in other cases reported in the literature (each number on the left corresponds to the bibliographic reference)

In our case and six other cases with ring chromosome 4 [3, 40, 42, 44, 48, 51], array-CGH was used to delimit the loss. However, only in our case and the other four cases [3, 42, 44, 48] has the loss been identified in 4p with an average size of 975,315 bp (range, 130,153-1,710,458 bp), and in this and two other cases [40, 48], the loss in 4q has an average size of 3,531,973 bp (range, 2,449,000-5,090,342 bp) (Fig. 6).

The difference in whether detecting deletions and variations in the deletion limits could be due to the type of array platform used and its resolution degree. The arrays utilized in this study and in other cases with ring chromosome 4 have a range of coverage between 44 K and 750 K.

The deleted region of 1,710,458 bp of 4p16.3 contained 34 genes. Of these genes, the following should be highlighted: phosphatidylinositol glycan anchor biosynthesis class G (*PIGG*). Allelic variants of this gene have been associated with intellectual disability, hypotonia, and early-onset seizures. Complexin 1 (*CPLX1*) gene positively regulates a late step in exocytosis of various cytoplasmic vesicles, such as synaptic vesicles and other secretory vesicles [58]. Fibroblast growth factor receptor-like 1 (*FGFRL1*) is a member of the fibroblast growth factor receptor (FGFR) family. Mice with a targeted deletion of the *FGFRL1* gene die perinatally due to alterations in their diaphragm. These mice also show bilateral kidney agenesis, suggesting an essential role for *FGFRL1* in kidney development. A human patient with a frameshift mutation exhibits craniosynostosis, suggesting an additional role of *FGFRL1* during bone formation [59]. The C-terminal binding protein 1 (*CTBP1*) gene encodes a phosphoprotein that is a transcriptional repressor and may play a role during cellular proliferation. Diseases associated with *CTBP1* include hypotonia, ataxia, developmental delay and tooth enamel defect syndrome [58].

In the other cases studied by array, the number of genes involved in the deletion was according to the size of the loss: six genes in the 130,153 bp deletion [48], 14 genes in the 759,758 bp deletion [42], 16 genes at the deletion of 897,434 bp [44] and 31 genes at the deletion of 1,378,772 bp [3].

In our study, the loss of 3,056,579 bp of 4q35.2 contained seven genes. These genes do not seem to be associated with the patient’s phenotype [58]. Previously, an interstitial deletion of 5.75 Mb has been described in the genomic region 4q35.1-q35.2 between 184,717,878 and 190,469,337 bp without discernible clinical effects [60]. In other cases, proportional to the size of the loss in 4q35, four genes were in the deletion of 2,449,000 bp [48], and 19 genes were in the deletion of 5,090,342 bp [40].

The clinical variability observed in our case and all the cases reported could respond to the size of chromosome 4 deletion involved in the ring as described for rings in other chromosomes [61, 62] and, for our case, the coexistence of the deletion and duplication of chromosome 4, which have rarely been reported in the literature.

## Supplementary information

**Additional file 1.** Comparison of clinical characteristics between the proposita and the reported cases with ring chromosome 4.

**Additional file 2.** Comparison of cytogenetic results between the proposita and the reported cases with ring chromosome 4.

## Data Availability

All data used in this study are available from the corresponding authors for request.

## Abbreviations

*ABCA11P*: ATP Binding Cassette Subfamily A Member 11, Pseudogene
APGAR: Appearance, Pulse, Grimace, Activity, and Respiration
*ATP5I*: ATP Synthase Membrane Subunit E
*C4orf42*: Chromosome 4 Open Reading Frame 42
*C4orf48*: Chromosome 4 Open Reading Frame 48
*CRIPAK*: Cysteine Rich PAK1 Inhibitor
*DGKQ*: Diacylglycerol Kinase Theta
*FAM53A*: Family With Sequence Similarity 53 Member A
*FRG1*: FSHD Region Gene 1
*FRG2*: FSHD Region Gene 2
*GAK*: Cyclin G Associated Kinase
*IDUA*: Alpha-L-Iduronidase
*KIAA1530*: UV Stimulated Scaffold Protein A
*LETM1*: Leucine Zipper And EF-Hand Containing Transmembrane Protein 1
*LOC100129917*: Uncharacterized LOC100129917
*LOC100130872*: Uncharacterized LOC100130872
*LOC401164*: Uncharacterized LOC401164
*MAEA*: Macrophage Erythroblast Attacher
*MFSD7*: Major Facilitator Superfamily Domain-Containing Protein 7
*MIR943*: MicroRNA 943
*MYL5*: Myosin Light Chain 5
*NAT8L*: N-Acetyltransferase 8 Like
*PCGF3*: Polycomb Group Ring Finger 3
*PDE6B*: Phosphodiesterase 6B
*POLN*: DNA Polymerase Nu
*RNF212*: Ring Finger Protein 212
*SCARNA22*: Small Cajal Body-Specific RNA 22
*SLBP*: Stem-Loop Binding Protein
*SLC26A1*: Solute Carrier Family 26 Member 1
*SPON2*: Spondin 2
*TACC3*: Transforming Acidic Coiled-Coil Containing Protein 3
*TMED11P*: Transmembrane P24 Trafficking Protein 11, Pseudogene
*TMEM129*: Transmembrane Protein 129
*TMEM175*: Transmembrane Protein 175
*TRIML1*: Tripartite Motif Family Like 1
*TRIML2*: Tripartite Motif Family Like 2
*TUBB4Q*: Tubulin, Beta Polypeptide 4, Member Q, Pseudogene
*ZFP42*: ZFP42 Zinc Finger Protein
*ZNF141*: Zinc Finger Protein 141
*ZNF595*: Zinc Finger Protein 595
*ZNF718*: Zinc Finger Protein 718
*ZNF721*: Zinc Finger Protein 721
*ZNF732*: Zinc Finger Protein 732
*ZNF876P*: Zinc Finger Protein 876, Pseudogene
